# Machine vision situations: Tracing distributed agency

**DOI:** 10.12688/openreseurope.16112.1

**Published:** 2023-08-31

**Authors:** Marianne Gunderson, Ragnhild Solberg, Linda Kronman, Gabriele De Seta, Jill Walker Rettberg

**Affiliations:** 1 Department of Linguistic, Literary and Aesthetic Studies, University of Bergen, Bergen, 5020, Norway; 2 Department of Art and Media Studies, Norwegian University of Science and Technology, Trondheim, 7491, Norway

**Keywords:** machine vision, digital humanities, agency, science fiction, games, art

## Abstract

This article proposes a new method for tracing and examining agency in heterogeneous assemblages, focusing on the role of machine vision technologies in creative works. We introduce the concept of the “machine vision situation”, defined as the moment in which machine vision technologies come into play and make a difference to the course of events. By taking situations as the unit of analysis, we identify moments at which machine vision technologies take part in actions without reducing them to either tools or protagonists, instead allowing for more complex agential entanglements between human and non-human actors. Grounded on an interdisciplinary theoretical framework, this article demonstrates how an analytical unit such as the machine vision situation is a valuable method for tracing distributed agency. We illustrate this through three examples by applying the method to creative works – narratives, digital games, and artworks – revealing key aspects of distributed agency and calling attention to the excess, complications, and messy entanglements that might otherwise be overlooked in analyses of agential assemblages. The machine vision situation is shown to be a method-agnostic unit of analysis that can be productively incorporated in both quantitative and qualitative studies and applied to other contexts in which human and non-human actors interact.

## Plain language summary

Machine vision – the ability of machines to “see” and interpret visual information – has advanced significantly in recent years, with applications ranging from self-driving cars to medical diagnosis. However, there is a growing recognition that this technological advancement is not simply a tool, but rather results in new distributions of agency alongside (and, at times, against) human decision-making.

Our article explores this idea in depth, examining how machine vision systems and human beings can be understood as agents in works of narrative such as games, art, and fiction. Analysing the representation of machine vision in artistic works reveals how these technologies are experienced and imagined in different contexts. The authors introduce the framework of “machine vision situations” to analyse the complex and dynamic relationships between humans and machines in both fictional and real-world contexts.

A machine vision situation is a moment at which a machine vision technology is seen to make a difference to the course of events. This situation can be analysed by identifying the actors involved and making a list of verbs that describe each of their contributions to the event. This method results in a dataset that can be analysed quantitatively, but it is also a generative starting point for a qualitative analysis of distributed agency between human and non-human agents in both fictional and real-world situations.

## Introduction

Who creates the image when you type a prompt into an image generator like DALL-E or Midjourney? Is it you or the AI? A person is wrongfully arrested after being misidentified by a facial recognition algorithm – who is responsible: the police, the programmers, or the algorithm? How do we understand agency and responsibility when automated entities create, interpret, and act upon information? These types of questions are gaining relevance as our lives become increasingly entangled with technologies that see, interpret, and make decisions with various degrees of independence. This paper proposes a model for analysing agency as it is distributed between human and non-human actors in what we call *machine vision situations*. To develop this model and demonstrate its potential, we use examples from fiction, games, and artworks. As machine vision technologies are popularised, the implications of these developments are explored in creative works of art, science fiction, and popular culture. Examining how machine vision is represented in these works reveals how the agency of these technologies is felt and imagined.

The notion of non-human agency has come to the forefront in recent decades in a wide range of theories and approaches that cluster under the terms posthuman theory, new materialism, affect theory, and science and technology studies (STS)-based approaches ( Bennett, 2010; Hayles, 2017; Latour, 2005; Massumi, 2021). Our premise, which we explore throughout the article, is that agency is not an exclusively human capacity. To understand human interaction with our environment, we need to take account not only of human choices and actions, but also of the agency of other lifeforms such as plants, animals, and microorganisms, as well as the agency that is expressed by non-living matter such as technical objects, weather phenomena, and other “things” ( Hayles, 2017). Acknowledging the agency of the non-human world undermines the position of the (hu)man as the sovereign actor who controls and transforms nature through the deliberate use of tools. What used to be a passive backdrop for human heroism comes to life, and the stage is cluttered with agents, allowing new dramas to play out. Through an analysis of the figurations of machine vision technologies in artistic works, this paper presents a method for bringing these dramas to light.

Automated systems and machine learning models are becoming prominent examples of these new configurations of agency. This paper focuses on the interventions and differences made by technologies that fall under the umbrella term of machine vision. Machine vision, understood as “the registration, analysis, and representation of visual data by machines and algorithms” ( Rettberg *et al.*, 2019, 1), brings us sights (the inside of one’s colon, the travel patterns of a population under lockdown, or a live feed of owl hatchlings huddled in a bird box) we would never have been able to see without technological mediation. The history of machine vision can be traced back to the invention and popularisation of the camera. It can arguably be extended to include the history of glasses, binoculars, microscopes, and other noteworthy early examples of technologies that have enabled us to surpass the limitations of embodied human sight. What is new is the extent to which machine vision reaches into our lives. Augmented by computational automation, technologies that fall under the umbrella of machine vision keep multiplying, encompassing widely different objects and uses, ranging from surveillance cameras and webcams to facial recognition and cancer detection. As an extension of these, there are neural networks and machine learning models that can generate new images based on existing images and datasets. Examples include deepfakes, computer-generated imagery (CGI) in movies, AI image generators such as DALL-E and Midjourney that generate images from written prompts, and amusing or pleasing social media filters.

How can we understand new distributions of agency between human and non-human actors, such as machine vision systems? How can we identify the contributions of each agent without losing track of their connectedness? Is it possible to design a method for looking at distributed, variously empowered agencies? How are human and non-human actors affected or changed in interactions with machine vision?

In order to investigate these questions, we set out to study how machine vision technologies figure in a wide range of artistic works. By investigating how machine vision is represented in art, games, and fictional narratives, we can learn how these devices figure in our collective imagination and how they are seen to meaningfully interact within societies. The phrase “meaningfully interact” is a key term here. In studying the distribution of agency among various actors, human and otherwise, it is essential to find methods that do not operate with the implicit assumption that agency is a zero-sum game, in which the agency of one actor automatically diminishes or cancels out the agency of others, or that there is some sort of binary opposition between human and non-human actors. Drawing on Lauren Berlant’s use of the situation as an analytical tool, we propose the concept of machine vision situations as a methodological lens to open up the analysis of human-machine interactions beyond the binary.

The concept of the machine vision situation was developed as we worked on the *Database of Machine Vision in Art, Games, and Narratives*. Completed in 2021, the database documents 77 digital games, 190 digital artworks, and 233 movies, novels, and other narratives that use or represent machine vision technologies ( Rettberg *et al.*, 2022a; Rettberg *et al.*, 2022b). It contains a total corpus of 500 works and, within these works, we have identified and analysed 874 specific situations where machine vision is central ( Rettberg *et al.*, 2022a; Rettberg *et al.*, 2022b). We defined machine vision as “the registration, analysis and representation of visual information by machines and algorithms” ( Rettberg, 2017), and based on this definition and an initial survey of creative works, we developed a list of 26 different technologies that formed the basis for identifying machine vision situations in the works. ^1^ Our goal in developing the database was to draw on the computational powers of the digital humanities to conceptually map commonalities and tendencies in representations of machine vision across a wide range of works, and to observe meaningful tendencies and connections which would not otherwise be apparent. This is a common approach in the digital humanities (see, for instance, Sinclair & Rockwell, 2015, 288). While many digital humanities projects rely on machine reading and interpretation of a corpus, our project relied on “manually” selecting the works to include in the corpus. The database structure functioned as a lens through which we constructed a body of data that would then be analysed using digital tools. However, the actual data resulted from each researcher’s reading and interpretation of the works. The process thus combines classical humanistic, interpretative approaches to text with digital, quantitative methods.

We have begun to publish findings from our analyses of this dataset, identifying what the most common technologies are doing in the works in which they are used or represented, and what is most commonly being done to them ( Rettberg, 2022b). For instance, drones are most commonly represented as recording, killing, transmitting and targeting and are represented as being controlled by human beings ( Rettberg, 2022c). Based on a machine learning analysis of the machine vision situations dataset, Rettberg (2022a) developed the method of ‘algorithmic failure’ to identify particularly salient cases for further study. The data collected on digital games formed the basis for a study on the use of surveillance cameras as an interface in digital games, proposing the term ‘cyborg vision’ to account for the experience of embodied surveillance that these games offer to the player ( Solberg, 2022a); a paper on how holograms mediate between human and non-human actors in games ( Solberg, 2021); and a paper on enhanced vision in games and its relation to ideas of domination and power ( Solberg, 2022b). The data collected on artworks formed the basis for Kronman’s (forthcoming) analysis of different approaches to hacking machine vision in art, revealing how art is used to expose bias in machine vision, as well as papers on non-conscious cognition and agency ( Kronman, 2020), and aerial perspective and prediction in machine vision assemblages ( Kronman, 2019). Gunderson (2021) drew on the database to analyse the representation of augmented reality in popular culture. While each of these papers grew out of the quantitative work that went into making the database, several have turned to qualitative, close readings of works and situations as their main analytical method. Based on this experience, this paper seeks to demonstrate how the concept of machine vision situation, and the formal data structure we developed for it, can serve as a tool to identify the compelling and relevant moments for analysis within a work, and function as a framework for qualitative analysis of distributed agency. As such, it offers the theoretical basis for and a description of the machine vision situation as an analytical model, a model we argue is broadly applicable well beyond studies of machine vision or digital methods.

Through the process of developing the analytical model with which to characterise the various ways that humans, machines, and other agencies are expressed in a wide range of works, we came up with the machine vision situation as a core unit of analysis. This allowed us to create structured data about the distribution of agency in machine vision interactions in a form that could be analysed quantitatively. Furthermore, as we will demonstrate in this paper, the situations model is a remarkably productive tool for qualitative analysis.

This paper focuses on the machine vision situation as a method and analytical tool, discussing its relation to different approaches to non-human agency, and demonstrating its potential for both quantitative and qualitative analysis of distributed agency. In what follows, we will trace how we developed the analytical model of machine vision situations and discuss how it informs and is informed by multiple approaches to non-human agency, drawing on Bruno Latour’s actor-network theory, N.K. Hayles’ concept of nonconscious cognition, and Lauren Berlant’s and Brian Massumi’s approaches to affect. This discussion is followed by three examples of machine vision situations from the database, illustrating how this tool can be used to analyse narrative works, games, and artworks. Finally, we will discuss how this tool may be used beyond the context of creative works and point towards their further development.

## Machine vision situations

Our goal was to understand interactions between machine vision technologies and other agents across a wide range of creative works: digital artworks, movies, digital games, novels, and more. To see patterns across genres and technologies, we needed a classification schema that allowed us to find shared characteristics in a very diverse dataset. The project necessitated the collection of representations of machine vision technologies from a large number of works emerging in disparate media and genres. Finding a format for analysis that allows for direct and easy comparison between the works was a challenge that required the design of a structure through which we could extract comparable meanings from, for example, a digital art installation, a digital game, a movie, a novel, and a piece of digital fiction. In order for works to be registered in the database, there must first be a conceptual infrastructure ( Feinberg, 2017) within which to register them. This process involved deciding on the categories under which works would be classified. In our case, this meant negotiating and discussing which aspects of the works had relevance for the project and how to capture them through keywords or predetermined categories. It involved deciding on the basic units of meaning through which to build a corpus of data that would lend itself to network analysis by making some features of the text itself explicit, with the goal of making them processable by some computerised application (Renear, 2004 in Pierazzo 2015:307). Computational methods of analysis have often been placed in opposition to “the individuated and situated practices of human reading and interpretation” ( Drucker, 2017, 631), but as Drucker points out, this is a false dichotomy. As Jill Walker Rettberg argues, “data is always partial and situated” ( Rettberg, 2020, 4), and designing a database or a text analysis program is an interpretative act ( Drucker, 2017), something the lively discussions within our research group can attest to. Designing a system in which to characterise often complex works through a limited number of keywords is a process of making careful trade-offs between specificity and generalisability.

We addressed this problem by developing the concept of *machine vision situation*, which we define as the moment at which machine vision technologies come into play and are seen to make a difference in a work. We give example readings of situations in Annalee Newitz’s short story *Drones Don’t Kill People* (2014), the computer game *Detroit: Become Human* (2018), and Anna Ridler’s digital artworks *Myriad (Tulips)* (2018) and *Mosaic Virus* (2019): in this section we outline the theoretical background and framework for the model.

A situation, according to Lauren Berlant, is “a state of things in which something that will perhaps matter is unfolding amid the usual activity of life” (2011:5) and “a genre of living that one knows one’s in but that one has to find out about, a circumstance embedded in life but not in one’s control” (2011:195). This concept of the situation as a moment of disturbance in everyday life that “forces one to [...] become interested in potential changes to ordinariness” (2011:195) is a very fruitful concept with which to analyse the cultural imagination of emerging technologies, such as machine vision. The task is to identify the situations in which machine vision technologies come to matter in the action of what is unfolding within or through the work. A machine vision situation may be an excerpt from a novel, a scene in a movie or a sub-plot in a game, or it may encompass a whole work, as may be the case with short stories or artworks – what we were looking for was the moments at which machine vision technologies were taking part in the action, so to speak. Each work may contain one or more of these situations in which one or more machine vision technologies come into play.

By defining machine vision situations as a core unit of the database, we identified moments where machine vision technologies became what Bruno Latour would call “matters of concern” ( Latour, 2004). In *Reassembling the Social* (2005), Latour argues that accounts of social agency are limited by their reliance on a conception of the social which precludes the inclusion of anything, or any *thing*, that is not composed, collectively or individually, of people. This social realm is seen as separate and clearly distinguishable from other realms of reality, such as physics, biology, or geology. In actor-network theory, which Latour developed in the 1980s and 1990s with Madeleine Akrich, John Law, Michel Callon and others ( Akrich, 2023), the social is redefined as “a type of connection between things that are not themselves social” ( Latour, 2005:5) and the task of the social scientist is redefined as “the tracing of associations between heterogeneous elements” ( Latour, 2005:5). Action, in the actor-network approach, is no longer limited to that which is intentional or meaningful human behaviour, but may just as well rest in “the domain of ‘material’ ‘causal’ relations” ( Latour, 2005:71). If something, or *some thing* “makes a difference in the course of some other agent’s action” ( Latour, 2005:71), that thing has agency within the context of which it is seen to matter. Building on this, the idea of machine vision situations is that looking at the doings of humans and technologies together allows us to see the difference each agent makes in the situation and to understand the specificity of their interactions. While actor-network theory has developed its own methodological tools for studying the interrelations of human and non-human agency, for our purpose we needed to develop a system that would allow us to generate data that could be studied quantitatively and which was suited to analyse works of fiction, games, and art. In order to capture these interactions within the various works, we set out to establish a model with which to encode the actions and actors involved that was simple enough to use as the structure of our database.

This brings us to our second major challenge: how to capture meaningful interactions across a wide range of machine vision technologies. Describing the agency expressed by a specific machine vision technology within a work was complicated by our realisation that machine vision technologies are often presented as doing many different things that cannot be easily reduced to a single entry. It was clear that we could not form our analysis of machine vision simply around the act of seeing. Some actions might more easily be described as generating, classifying, or identifying, but even that seemed insufficient to capture what these devices were doing in our lives on a meaningful level. The same technology may do different things in different contexts or different works. However, it is easy to take the agency of non-organic objects as given by their known functions and uses. As Massumi states, “a thing is when it is not doing” (2021:7), meaning that when an object is not doing, it is fixed, known, static – but we are interested in the doing, the undetermined potentiality for action, and their ability to cause something to happen, without assuming to know in advance the difference they would make. Instead of looking at machine vision technologies in their “thingliness”, as objects with known uses and fixed functions, we set out to study their manifold actions as embodied and/or articulated in a wide range of works. As a result, we decided to describe what technologies and other actors are doing in each situation through an open vocabulary of verbs. We landed on a system in which we registered one or several machine vision technologies and any characters and/or entities that were active in each machine vision situation. ^2^ For the purpose of this paper, it is sufficient to describe characters as people or other creatures for whom we want to register secondary characteristics, such as gender ^3^, age, etc. Entities are objects, institutions, or generic categories such as “users” or “images” for which gender or similar characteristics cannot be identified. Each agent (a technology, character, or entity) was then assigned a verb to describe their interaction with the other agents in the situation. The analyses of machine vision situations later in this paper demonstrate how this works in practice. By attributing verbs to machine vision technologies, they become visible as agents within these situations. Suddenly, their active participation in a chain of events comes to the foreground. This enabled us to capture some of their liveliness, while remaining open to the possibility that technologies, people, and other entities may be seen to be doing several things at once, without predetermining the types of actions that may be taken by any of the actors involved. Following Latour (2005), in order to understand the unfolding of social (and non-social) events, the contributions of each element in the chain, or network, of action must be taken into account: the researcher should trace the connections that enable social agency, through a network composed, as it may be, of objects, groups, documents, and people. In the case of the machine vision database, the verbosity may be an apt method to trace the difference these technologies make as linguistic markers of our vibrant relationship with that which we are used to thinking about as matter, resources, or tools.

Studying humans, technologies, and other entities as potentially lively “bodies with the power to affect and be affected” ( Massumi, 2021:16) in unforeclosed ways also required a system for capturing the effects other actors have on each entity involved in a situation. We used the present continuous tense (e.g. *watching*) for actions undertaken by a character, technology, or entity, and the past participle as used in the passive tense (e.g. *watched*) for actions that happen to a character, technology, or entity. This enabled us to identify the various activities that machine vision technologies are involved in, as well as their effects on the various actors involved. This inclusion of passive verbs reveals how the various agents in the situation are affected by each other’s actions, bringing out how they are both being changed and effecting change on other bodies within the situation.

We made a deliberate choice to position technology, people, and institutions on the same level in the machine vision situation. Our analytical model has no predetermined structure that pre-defines technologies as passive, or as tools or objects, or that positions humans as users, creators, or subjects. This even-handed treatment allows both machines and people to be assigned agency in a given situation. The tagging system, by design, prescribes a reading of these technologies as active agents. As such, it could be criticised for imposing a top-down, theory-informed interpretation onto the material. However, to be able to see technologies’ contribution to agency, they first need to be seen as possible agents. A deliberate step away from their automatic classification as passive objects and mere tools is necessary to access that perspective. Only once that step has been taken are we able to see the ways in which technologies make a difference as “participants in the course of action” ( Latour, 2005: 71).

In the database, however, the technologies, as well as the characters and entities, appear as separate, predefined categories. This is a departure from the actor-network-theory approach of allowing the subjects to appear as you trace their interactions. By designating them with controlled vocabularies, the actors appear more fixed and stable than they might have any reason to be. The limitations of the understanding of agency imposed by the database structure are particularly apparent when seen through the lens offered by Jane Bennett in her book *Vibrant Matter*. Building on Latour, she theorises non-human agency through the Deleuzian concept of assemblages, “ad hoc groupings of diverse elements” ( Bennett, 2010:23) through which agency is effectuated and unfolds. In *Vibrant Matter*, she highlights the active participation of non-human matter in public life through the concept of thing-power to describe “the material agency of natural bodies and technological artifacts” ( Bennett, 2010:xiii). Analysing the Northeast blackout of the electrical power grid in the US in 2003, she de-emphasises the role of the individual subject (or object) in the incident that affected around 55 million people. She argues that agency is not so much the result “of a doer (an agent) behind the deed (the blackout) as a doing and an effecting by a human-nonhuman assemblage” (2010:28), composed of “a heterogenous series of actants with partial, overlapping, and conflicting degrees of power and effectivity” (2010:33). Agency, for Bennett, is not controlled from one central node; rather, the ability to make something happen may arise from the emergent properties of assemblages of matter, bodies, and forces. The machine vision situation is our attempt to map some of these assemblages and trace the agency as it emerges between and among the diverse bodies involved. The verbs attached to the various agents highlight the distribution of agency throughout the assemblage, bringing out their diverse contributions of causes and effects while demonstrating their interconnectedness.

So, what is being done in these human-non-human assemblages? How do technologies such as facial recognition, satellites, or drones figure in these assemblages? What kind of action do they effect? This is where N. Katherine Hayles’ concept of nonconscious cognition is useful. In *Unthought*, Hayles proposes a definition of cognition that allows us to understand technological objects as cognisers: “ *Cognition is a process that interprets information within contexts that connect it with meaning*” (22; emphasis in the original). She argues that while consciousness may be attributable primarily to humans, cognition is a capacity that we, according to Hayles (2017), share with other animals, perhaps even plants, as well as with technical devices. Hayles describes computational media, and especially AI, as “quintessentially cognitive technologies” ( Hayles, 2017:41) with the ability to process information, identify patterns, and make inferences. When we use these technologies, we effectively enter into what Hayles terms “cognitive assemblages,” which, for the example of a cell phone, would include “relay towers and network infrastructures, including switches, fibre optic cables, and/or wireless routers, as well as other components” ( Hayles, 2017:8). When we seek to describe what machine vision technologies are doing, their interpretative and meaning-making contributions are readily apparent.

To sum up – in order to study what is being done with, by, and to machine vision technologies, we begin by identifying situations in which these technologies are seen to make a difference. We then identify the agents involved in the situation – the specific technologies, characters, and other entities participating in the action. The next step is to look closely at the situation, and attribute verbs to each of the agents involved, based on what they are seen to be doing, using passive verbs to describe the effects of actions, if appropriate. The verbs should be allowed to emerge from the text(ure) of the situation, and the same agent may have several different, even contradictory, verbs attached to it. Relatedly, the verbs are not limited to the supposed or assumed uses or activities in which a body or object of its kind is assumed to take part.

In what follows, we will go through machine vision situations from three different works: a short story, a computer game, and an artwork, to demonstrate how this analytical model can be applied to explore the representation of agency in a variety of artistic works.

## Assemblages in the works

### Drones Don’t Kill People

The result of this analytical model is a snapshot of actions attached to each actor, which can, on their own, form remarkably compelling narratives. For example, in the short story *Drones Don’t Kill People* by Annalee Newitz (2014), narrated from the perspective of an artificially intelligent surveillance drone, one machine vision situation occurs when a group of largely autonomous AI drones, subcontracted to a corporation working for the Turkish government, are set to monitor a professor and his family and to pass on potentially relevant information. One night the drones observe the unnamed mother answering a question posed by the daughter. This is what the incident looks like in the database:

*Professor’s Daughter: is Questioning*
*Professor’s Wife is: Answering, Explaining, Revealing*
*Drones are: Spying, Recording, Interpreting, Selecting, Debating*
*Corporation is: Subcontracting, Employing, Surveilling, Deciding*

Here we see that although the drones perform expected machine vision actions such as recording, they are also partaking in other more cognitive and collaborative activities involved in the same process. The inclusion of “spying” furthermore emphasises that what they are doing has normative implications in a larger social context. The mother and daughter’s actions are linked to the drones’ activity through the inclusion of “revealing”. Finally, the corporation’s actions are included in the assemblage, highlighting that the drones are not acting in a vacuum. The corporation is the agent attributed with the action of “surveilling” since they, not the drones, decide what to do with the information which is collected. Together, they enter into what Bennett (2010) might describe as an assemblage of machine vision-mediated activity. However, this does not mean that agency is equally distributed among the actors, which will become evident in the next entry from the same short story.

Based on the information collected from this incident, the professor and his family are identified as political dissenters and activists, and the drones are ordered to assassinate them. In the database, the entry for this second situation looks like this:

*Corporation is: Subcontracting, Employing, Deciding, Ordering*
*Drones are: Obeying, Deciding, Killing, Watching, Recording.*
*Professor is: Killed*
*Professor’s Wife is: Killed*
*Professor’s Son is: Killed*
*Professor’s Daughter is: Evading, Watching, Screaming, Killed, Recorded.*

Both the drones and the corporation take part in the act of killing the professor and his family. The unequal distribution of power between the drones and the corporation is made explicit through the inclusion of the verbs “ordering” and “obeying,” while the inclusion of “deciding” emphasises the drones’ active role. Here, the act of killing is attributed to the drones, although this will be contested later in the story, as foreshadowed by the title. The assassination went according to plan, although it did include one “statistically anomalous event” when the drones missed the daughter on the first shot. Her screams of terror as she watched them kill her family were recorded in their distributed memory. The way this situation is written, the family, with the exception of the daughter, have very little agency, as their lives are extinguished before they can register what is happening or respond – as Bennett would say, while the drones are acting, they are “suffering action” (2010:21). The daughter suffers the same fate, but her final actions reverberate throughout the story – through the recording of the incident, her screams will continue to have the power to affect the drones.

The act of assigning the actors and verbs involved in a situation is inevitably interpretative, and revisiting these entries inevitably brings up potential edits and additions that could have been made. For instance, rereading the short story revealed a third situation, overlooked at the time of data entry, but readily apparent in the light of this paper: after the contract with the Turkish company expires, the drones are subcontracted to the Uyghur Republic ^4^ government to monitor a desert highway in contested territory bordering on China. Little of relevance is happening, and for the first time, the drones are left alone with nothing to do, so they look about on the web for stuff to analyse. As drones, they are programmed to recognise faces, so when they come across images of drones with faces painted on them, they begin investigating their own identity.

*Government is: Ordering, Surveilling, Ignoring*
*Drones are: Bored, Analysing, Sharing, Recognising, Changing*
*Images are: Fascinating, Recognised*

In this situation, images appear as an agent for their capacity to capture the drones’ attention and elicit a moment of recognition and interest in themselves as drones. Here, something unexpected occurs: the drones take the first step towards self-awareness and, thus, consciousness. As a result, they become interested in themselves as agents in the world. This is the true turning point in the story, taking the drones down the path of forming a political movement fighting for the rights of AIs. The images are clearly making a difference to the drones’ course of action in the Latourean ( Latour, 2005) sense, and in this assemblage, they are the pivotal actors.

In all three of these situations, something new emerges in the machine vision assemblage. In the first one, the ambiguity of the information makes the drones debate whether or not to include it in their report. In the second situation, the recording of the screaming daughter will reverberate in the drones’ collective memory. In the third situation, the moment of self-recognition prompts the emergence of self-awareness, opening the drones up to change and their emergence as political subjects. The assigned verbs do not necessarily capture these becomings as they are matters of affect, not action – they are what takes form beyond the actions taken by each of the entities of the situation, changes in positionings, and the seeds of future events ( Massumi, 2021). Nevertheless, tracking these changes can point to moments where action and affect come together to make something new. This would not become apparent in a quantitative analysis of a great number of machine vision situations like the ones collected for the database. However, each situation is an invitation to look closer, follow the connections and interactions that become apparent through this method, and look for what emerges beyond the verbs.

Registering incidents in a short story using this framework may be relatively straightforward. However open to interpretation and shifting meanings, a text is still a relatively static object in the sense that the words appear in the same order every time you encounter them, and the narrative structure easily lends itself to the process of identifying actors and actions. In what follows, we will show how machine vision situations work when considering other genres, looking first at a game and then at two works of art.

### Detroit: Become Human

The doing can take on various forms across media. Audiovisual media like films and digital games often present actions visually, and in most games, this visualisation will also require some sort of haptic action from its user. Using situations to trace agency in games shows these various entanglements of action and bodies across levels of virtuality.

Consider the game *Detroit: Become Human*, published in 2018 by Quantic Dream, which follows androids as they navigate their emerging sentience in a society that places them in positions of servitude and submission. One of these androids is the police inspector Connor, whose job is to find and capture rogue sentient androids. Due to Connor’s android body, the presence of various machine vision technologies embedded in his body is unquestioned. Connor’s augmented vision includes an augmented reality overlay of the world, combined with object recognition technology, vision beyond the human spectrum, and even reconstructive/generative image software that can visualise past events leading up to the crime scenes before him. These technologies perform various actions, but are combined in the character of Connor, played by the player. Connor’s actions are also actions on behalf of the game software, the video game console, the player, and various machine vision technologies, showing the interdependence and permeable boundaries between various agents in the assemblage ( Bennett, 2010). This method also highlights the challenge of determining which actors are registered and are thus made visible. For our purpose, we decided to solely include the actors represented within the work, which means that the database does not register the player or the console here, but the combined action of distributed agents, as represented by Connor.

In one situation, two guards escort Connor into a corporate elevator to present him to their boss. Once inside the elevator, a perceptive Connor/player can identify a surveillance camera in the top corner, hack into it, kill the two guards, and escape without repercussions. The situation entry looks like this:

*Connor is: Hacking, Fighting, Cloaking, Killing*
*Surveillance camera is: Hacked, Blinded*
*Law enforcement is: Fighting, Killed*
*Corporation is: Blinded*

Here, we see that the entry creates a directional relation between Connor’s hacking and the surveillance camera that is hacked. In turn, Connor’s hacking not only blinds the surveillance camera, but also the corporation to which it belongs. The surveillance camera becomes a Latourean ( Latour, 2005) “thing” that influences another agent’s action. In assigning the same passive action of “blinded” to both surveillance camera and corporation, a temporal relation is created between the two. This relation emphasises how the surveillance cameras are the prosthetic eyes of the corporation in this situation.

Many games offer diverging and possibly excluding narrative paths, and *Detroit: Become Human* is no exception, as it is structured around presenting influential narrative choices to the player. If Connor does nothing in the elevator, he will be shot and killed when the doors open again. If Connor attacks the guards in the elevator without disabling the surveillance camera, he will have to fight more guards upon exiting, because he will have been spotted acting deviantly, i.e. more sentient than the humans prefer. Connor can also fail at fighting the guards. Following this pattern of diverging paths, the elevator situation will not even occur in some playthroughs because it depends on previous choices.

The science fiction world of *Detroit: Become Human* exemplifies the characterisation of technologies. In this situation, Connor is the agent, not the various processes that combine into him. The player’s bodily labour of choosing and possibly failing at performing actions is hidden in the situation analysis. This reveals how human and non-human agencies combine or work in tandem beyond the fictional world. Even if the player is not an explicit agent in this situation, they are implicit in the presence of the player character’s actions. In a different context, the situation could be expanded to include the player, as well as the console, or even the company or programmers who made the game, and so forth – one could follow these connections forever. The boundaries of the assemblage must be drawn with concern for the specific purpose of the knowledge project of which it is a part.

## *Myriad* and *Mosaic Virus*

In art, situations allow multiple layers of analysis. Digital artworks can put the viewer in a situation where they interact with machine vision technologies. They can represent fictional or actual machine vision situations in the same way as games and narratives do. Digital artworks also often highlight their own creation, that is, the situation in which the artwork was created. Anna Ridler’s diptych *Myriad (Tulips)* (2018) and *Mosaic Virus* (2019a) comprises two of many artworks where artists use machine learning-based AI image generation to create art *. Myriad (Tulips)* exhibits a dataset of hand-labelled polaroids classifying – according to colour, type, and stripe – a myriad of tulips, that is, ten thousand tulips. AI image generation often appears to be an automated process, but as Ridler describes it, dataset curation involves an “insane amount of work and it is usually work that is hidden” ( Ridler, 2019c). To create the dataset, Ridler selected the tulips at the market, photographed them, and sorted and labelled them. Thereafter, Generative Adversarial Networks (GANs), a type of machine learning model, were trained with the dataset. What the network learned from the ten thousand images of tulips was to hallucinate “botanical impossibilities” ( Ridler, 2019b). In the second part of the diptych, *Mosaic Virus*, these generated images are displayed on screens that show how the AI-generated tulips evolve. As Ridler explains in her artist statement, the “tulips are controlled by the price of bitcoin”. The aesthetic choice of bringing AI, bitcoin, and tulips together combines the historical speculative bubble created by the 17th-century Tulipmania with contemporary speculative investments in AI cryptocurrencies.

The identified machine vision situation in the database looks like this:

*Creator (Artist) is: Classifying, Labeling, Selecting, Speculating*
*Machine learning, Image generation is: Learning, Generating, Hallucinating, Co-creating*
*Images are: Classified, Generated*

Little of the aesthetics of the artwork remain in the reduced situation entry; however, what is implied is a distribution of cognitive labour between the artist and the machine. The artist creates categories and then interprets photographs classifying the tulips for the datasets. In both Ridler’s classification of tulips and our analysis of this as a machine vision situation, “interpretive flexibility” occurs, despite rigid classification protocols ( Feinberg, 2017). Ridler notes that even “something as simple as a tulip is difficult to put into discrete categories – is it white or pale pink, is it orange or yellow”. In turn, the image-generating machine learning model learns to recognise patterns in the 10,000 examples in the dataset, in order to generate new images of tulips. While human agency is still crucial to create an AI artwork like *Mosaic Virus,* non-conscious cognition like discerning patterns and drawing inferences is externalised to machine vision.

The creation of AI art emerges out of cognitive assemblages involving both human and machine interpretation. However, when AI art was popularised by image generators like DALL-E and Midjourney producing new images from text prompts, it became easy to forget the human labour involved. The outcry from artists whose labour has been scraped into internet-sized datasets to train AI image generators highlights AI’s utter dependence on data created by humans ( Benzine, 2022). We can bring the human back into AI creativity through Hayles’ notion of “punctuated agency” that “operates within regimes of uneven activity, longer periods when human agency is crucial, and shorter intervals when the systems are set in motion and proceed on their own without direct human intervention” ( Hayles, 2017, p. 32). In the situation from Anna Ridler’s *Mosaic Virus*, the concept of “punctuated agency” helps us understand how the cognitive creation of an artwork is distributed between the artist and the machine: To create *Mosaic Virus*, the artist makes conscious aesthetic decisions when collecting and classifying a dataset, choosing a model, and adjusting parameters. The machine learning model then operates within these parameters. Hayles discusses how human non-conscious processes “feed forward intuitions to conscious awareness” (p.41); likewise, we can understand the technical non-conscious of machine learning models as generating a type of “technical intuitions” ( Kronman, 2020). In *Mosaic Virus,* technical intuitions are expressed as visual hallucinations of impossible tulips. Technical intuitions are then fed forward to conscious cognition when Ridler curates the generated images as an artwork and when the audiences of *Mosaic Virus* make sense of what the machines hallucinated. This demonstrates the back-and-forth activity of punctuated agency between human and machinic agents within the same assemblage. The specificity of punctuated agency between the actors is not explicitly registered within the machine vision situation structure. Again, however, the format functions as an entryway or invitation to look closer and uncover the connections and processes behind the activities brought out by the verbs.

These three analyses of situations from cultural works from different genres should hopefully give the reader some idea of the potential of the machine vision situation as a method. Although initially designed for the purpose of quantitative analysis across works, in the above three examples, the machine vision situation functions as the entry point for qualitative analysis of human-non-human assemblages. As demonstrated, simply identifying the actors and their doings in a situation produces new insights into how humans, technologies and other entities affect and are affected by each other in everyday interactions. This structure creates a rich foundation for closer analysis, inviting the researcher to trace the connections between the agents, understand the processes behind the verbs, and see what emerges from the situation as a whole.

## Tracing agency

This paper takes up the task of theorising how agency is distributed when human and non-human actors interact. It presents a new method for tracing and examining agency in heterogenous assemblages in specific situations. We did so by focusing on automated systems, in particular machine vision technologies, as they are represented in creative works. Our contribution to debates about the distribution of agency is the concept of the “machine vision situation”, which we define as “the moment in which machine vision technologies come into play and are seen to make a difference in a work”. This moment becomes the point of departure for a closer analysis which involves identifying the main actors, their actions, and their effects on each other. With the machine vision situation framework, our aim is to identify and analyse moments in which machine vision technologies take part in actions, without reducing them to either tools or protagonists, but allowing more complex entanglements between human and non-human actors. In order to theorise the machine vision situation and explain how this method serves to explore an expanded understanding of agency, we drew on the work of key theorists across disciplines: Lauren Berlant’s thoughts on the situation as a genre, Latour’s actor-network theory, Massumi’s ideas on affective bodies, Bennett’s concept of agentic assemblages, and Hayles’s work on non-conscious cognition.

We use the analytical model of the machine vision situations as a method to trace distributed agency. As shown in the three examples summarised in the second part of this paper, situations can be identified in different types of creative works (narratives, digital games, artworks), and the role that machine vision technologies play in situations can be analysed, tracing new distributions of agency. In *Drones Don’t Kill People* the situations helped us understand the distribution of power between the actors. Furthermore, they brought out the pivotal moments through which something new emerged out of human/non-human assemblages. In *Detroit: Become Huma*n, we saw how the agency as represented within the work calls attention to its entanglement with the agency that was not included in the situation – that of the player and the console and the game itself as a technical/digital object. With the artworks *Myriad* and *Mosaic Virus*, analysing the machine vision situation reveals how cognition is distributed between human and technical actors in machine learning processes, while also calling attention to what does not fit into the situation structure – the process of punctuated agency between them. In each of these works, the machine vision situation reveals core aspects of the distribution of agency. Furthermore, the situation functions as a provocation, an incitement to look closer at what does not fit the structure, the excess, the complications – and that is often where a more profound understanding is to be found. By following a set structure, messy entanglements that might otherwise have been overlooked call attention to themselves and become obvious.

Our conceptualisation of machine vision situations was integral to creating the *Database of Machine Vision in Art, Games and Narratives*. Taking the machine vision situation as the core unit of analysis allowed us to create structured data about the distribution of agency in machine vision interactions in a form that could be analysed quantitatively. As we have demonstrated in this article, the machine vision situation can also support qualitative analyses by allowing researchers to disentangle complex distributions of agency in individual moments when machine vision technologies make a difference. The machine vision situation is thus not just a digital humanities concept, but also a method-agnostic unit of analysis that can be used productively in both quantitative and qualitative studies.

While our project collected and analysed data about creative works, we are confident that the machine vision situation can be productively applied to other contexts in which human and non-human actors interact, including everyday encounters with machine vision and the discursive imaginaries through which people make sense of these technologies. Agency is distributed through action, interaction and narrativisation, and we expect that the framework of the machine vision situation can be applied similarly across disciplines and methodological approaches.

Finally, while we conceptualised it through the study of machine vision technologies, there is no reason why using situations to analyse agency should be limited to this technology. As a method, situations should be generalisable to any interaction between human and non-human actors in which agency is distributed in novel ways; hence, further theoretical work could expand it into a more general concept of a “sociotechnical situation”.

By describing what both technologies and humans do in each situation and attaching actions to each of them, we produce information on how they are represented as actors and how actions, or doings, are distributed between them. As a result, we are able to trace the interpretative, meaning-making and communicative contributions of machine vision technologies, as well as their material functioning. As such, we as researchers also constitute an assemblage with the database; the database emerging through the parameters we give it, and the database subsequently directing our actions, interpretations and readings. The concepts, theories and ideas that arise from it cannot be traced back to a single researcher or even be limited to our efforts as a team; the database itself has an active role in their creation. Our thoughts and ideas, and future articles, will contain traces of the effects of this assemblage.

The knowledge we produce is conveyed with and through the structure we created to produce it, but that does not mean that it is contained by it. In the process of creating a quantitative dataset, discussing the structure, reading, playing, interacting with works, and agonising over which verbs to assign, a method emerges and takes on a life of its own. Quantitative data unfolds and reveals itself as a qualitative wellspring.

## Ethical approval

Ethical approval and consent were not required.

## Data Availability

The underlying data has been deposited in the UiB Open Research Data repository DataverseNO, https://doi.org/10.18710/2G0XKN ( Rettberg *et al.*, 2022a). This dataset contains all the entries on narratives, games, and artworks in the Machine Vision Database. See Rettberg *et al.* (2022b) for a detailed description of the data. This project contains the following underlying data: 00_README.txt 01_codebook.csv 02_technologies_sentiments_topics_definitions.csv" creativeworks.csv situations.csv characters.csv narrativegenres.csv situation_description.csv situations_visual.csv creators.csv worksinfo.csv machinevisionscripts.R Data is available under the terms of the Creative Commons Zero “No rights reserved” data waiver (CC0 1.0 Public domain dedication).
