# Supplementary material for: Machine vision situations: Tracing distributed agency
Source: Open Res Eur. 2024 Mar 15;3:132. Originally published 2023 Aug 31. [Version 2] doi: 10.12688/openreseurope.16112.2 (PMC11036037; doi:10.12688/openreseurope.16112.2)
Supplement: Peer review history

# Reviewer report: Reviewer response for version 2

**DOI:** 10.21956/openreseurope.18792.r38965

**Published:** 2024-04-22

**Authors:** Anastasia Salter

**Affiliations:** 1 University of Central Florida, Orlando, Florida, USA

I assessed and approve the revisions.

If any, are all the source data and materials underlying the results available?

Yes

Is the work original in terms of material and argument?

Yes

Is the work clearly and cogently presented?

Yes

Is the argument persuasive and supported by evidence?

Yes

Does the research article contribute to the cultural, historical, social understanding of the field?

Yes

Does it sufficiently engage with relevant methodologies and secondary literature on the topic?

Yes

Reviewer Expertise:

NA

I confirm that I have read this submission and believe that I have an appropriate level of expertise to confirm that it is of an acceptable scientific standard.

# Reviewer report: Reviewer response for version 2

**DOI:** 10.21956/openreseurope.18792.r39522

**Published:** 2024-04-22

**Authors:** Kristin Veel

**Affiliations:** 1 University of Copenhagen, Copenhagen, Denmark

It has been a great pleasure to read this article which I think provides an important methodological contribution and presents a series of interesting analyses that will be of great value to further work in the field. The article aptly outlines “machine vision situation” as a theoretical concept and demonstrates its usefulness as an analytical tool which allows for the integration of distant and close reading methods in fruitful ways. It is of particular interest to see how the method works across literature, games and art. This scope allows for valuable considerations regarding how to address the presence of machine vision technologies in a text/artwork/game and focusing on its implications for the narrative/story world/aesthetics across different fictional genres.

 The authors have addressed the concerns raised by the previous reviewers and the article now stands as a thorough study that is innovative and thought-provoking both methodologically, theoretically and analytically.

 One minor typo:

 "The police, the algorithm, or its programmers?" is repeated twice in the introduction.

If any, are all the source data and materials underlying the results available?

Yes

Is the work original in terms of material and argument?

Yes

Is the work clearly and cogently presented?

Yes

Is the argument persuasive and supported by evidence?

Yes

Does the research article contribute to the cultural, historical, social understanding of the field?

Yes

Does it sufficiently engage with relevant methodologies and secondary literature on the topic?

Yes

Reviewer Expertise:

My research engages with the cultural implications of technologies in art, literature and film and I have for instance worked specifically on drones as well as machine vision aesthetics.

I confirm that I have read this submission and believe that I have an appropriate level of expertise to confirm that it is of an acceptable scientific standard.

# Reviewer report: Reviewer response for version 2

**DOI:** 10.21956/openreseurope.18792.r38964

**Published:** 2024-04-05

**Authors:** Susana Tosca

**Affiliations:** 1 University of Southern Denmark, Odense, Denmark

I have read the improved version of the article and I think that it satisfactorily addresses the concerns raised in my review and more. The authors have added essential context so that their contribution shines in their own right.

If any, are all the source data and materials underlying the results available?

No source data required

Is the work original in terms of material and argument?

Yes

Is the work clearly and cogently presented?

Yes

Is the argument persuasive and supported by evidence?

Partly

Does the research article contribute to the cultural, historical, social understanding of the field?

Yes

Does it sufficiently engage with relevant methodologies and secondary literature on the topic?

Partly

Reviewer Expertise:

I am a media researcher, with speciality in reception, audience studies, entertainment media.

I confirm that I have read this submission and believe that I have an appropriate level of expertise to confirm that it is of an acceptable scientific standard.

# Reviewer report: Reviewer response for version 1

**DOI:** 10.21956/openreseurope.17395.r34881

**Published:** 2023-11-03

**Authors:** Anastasia Salter

**Affiliations:** 1 University of Central Florida, Orlando, Florida, USA

I enjoyed reading this article and found the framing in humanities discourse (and particularly creative works) unusual for this type of work. This is a timely and compelling study of representations of machine vision technologies, with a particularly useful investigation of the agency given to non-human actors in the context of a range of media. The wide range of works included in the underlying dataset allows the authors to interrogate those representations with attention to how we categorize and understand technologies such as facial recognition, satellites, drones, etc in the context of “human-non-human assemblages.

 Given the current popular attention to machine vision related work (and particularly the intense reactions to image generators), this article’s close analysis of three works will be of particular use to those guiding conversations and mediating reactionary discourse. As an educator, I certainly will be drawing on this useful analysis of “Drones Don’t Kill People” for my own AI humanities classes, and I appreciate its inclusion here.

 From a game studies perspective, the only section that raised some concern for me was the discussion of Detroit: Become Human. This game’s representation of Black masculine identity and use of parallels between android segregation and US history specifically has been the subject of some significant work already (I’d particularly recommend Rebecca Leach and Marco Dehnert’s article from 2021). This analysis decontextualizes the human (and non-human) stakes of the agents by not engaging with these important aspects of embodiment, which is particularly critical to the author’s discussion of “a society that places them [androids] in positions of servitude and submission.” As Susana Tosca has already noted in the previous reviewer report, there’s also a positioning of the interactor here that I concur warrants further discussion.

If any, are all the source data and materials underlying the results available?

Yes

Is the work original in terms of material and argument?

Yes

Is the work clearly and cogently presented?

Yes

Is the argument persuasive and supported by evidence?

Yes

Does the research article contribute to the cultural, historical, social understanding of the field?

Yes

Does it sufficiently engage with relevant methodologies and secondary literature on the topic?

Yes

Reviewer Expertise:

Digital humanities, electronic literature, game studies

I confirm that I have read this submission and believe that I have an appropriate level of expertise to confirm that it is of an acceptable scientific standard, however I have significant reservations, as outlined above.

## References

[1] : Becoming Human? Ableism and Control in Detroit: Become Human and the Implications for Human-Machine Communication. *Human-Machine Communication* .2021;2: 10.30658/hmc.2.7 137-152 10.30658/hmc.2.7

# Response

**Published:** 2024-03-15

**Authors:** Marianne Gunderson

**Affiliations:** Linguistic, Literary and Aesthetic Studies, University of Bergen, Bergen, Hordaland, Norway

Dear Prof. Salter,

 Thank you for your constructive and helpful review. We appreciate your thoughtful feedback and found it very useful for our revision process. We have made the following changes based on your comments:

 As mentioned in our response to Tosca's review, we agree that much more can be said about player or user agency in games and art; their absence in our analyses was an unforeseen result of our choice of examples. We have added a paragraph to both the art and games sections emphasising this and indicating how the user or player could come into play, as well as when and why we chose to include or exclude the player or user as an actor in our analyses. Here, we also point towards how the boundaries for which actors to include must be redrawn according to the priorities and limitations of the specific research project and indicate what this may bring out in the resulting analysis. Per your suggestion, we also brought the context of race and embodiment in relation to Detroit:Become Human into this section, drawing on the article you recommended as well as a couple of others.

 With these revisions, we believe that we have addressed all your major objections and hope that this version will meet your expectations.

# Reviewer report: Reviewer response for version 1

**DOI:** 10.21956/openreseurope.17395.r34883

**Published:** 2023-09-28

**Authors:** Susana Tosca

**Affiliations:** 1 University of Southern Denmark, Odense, Denmark

I really enjoyed reading this article. It provides a valuable contribution to understanding the topic of machine vision and the way we consider media-human configurations in general (which they by the end call "sociotechnical situations"), so it also has methodological interest. The "Machine vision situation" as a productive idea that allows for more nuanced analysis than for instance a thematic analysis, it refer to instances not just where machine vision appears but where it becomes an issue, there are tensions, breakdowns, etc. The article provides an alternative way of articulating and analysing non human agency, something that STS literature not always achieves. It was useful and inspiring.

 I found the paper particularly strong in the way it shows that a combination of digital methods and qualitative analysis, can produce interesting results, and it fully convinces in its claim that designing a database is an interpretive art. The three analyses cases, although brief, also do a good job of show how machines seeing can be broken up in a lot more agentic verbs like generating, classifying, identifying…The examples are strong and well argued. I must admit that after reading the first example I thought: is this all there is to analyse? But then it struck me that the power of this method is not in the individual semantic depth, but the sum of all these agency oriented analyses in a huge database that can yield interesting results. Perhaps this cumulative aspect could be made more clear in the paper. In short, to expand/explain their claim that “Taking the machine vision situation as the core unit of analysis allowed us to create structured data about the distribution of agency in machine vision interactions in a form that could be analysed quantitatively”. I would also like to know more about how the analysis of fictional material can enrich our understanding of machine vision "in real life", as it is just mostly assumed it does, without an actual argument.

 I have also indicated above that there are possibilities for improvement, which I would recommend the authors address to review the paper. The first and most important criticism in a paper centered around analysing situations is that the method of situational analysis is not even mentioned. Adele Clarke has worked on this method (inspired by grounded theory) for many years now, starting a whole school of thought around it, with countless examples of analyses and applications. This omission is even more glaring given that Clarke is also inspired by STS and including technology and non human agency in her way of approaching situations. Some of the situation theorists she bases her method on are also absent here, like C. Wright Mill or Donna Haraway.

 The second important issue in my view is that in the discussion of computer games and artworks, the agency of the user/interactor is not a part of the analysis, which is limited to the, shall we call it, diegetic aspects of machine vision. But unlike in literature, users have to activate/manipulate machine vision in these assemblages. Can this be addressed somehow in the discussion?

 As a minor issue, the Introduction section is a bit repetitive. I think I counted three times of similar expressions to "we came up with the concept of the machine vision situation". It can be tightened up. Also, the "previous literature" part seems to be a bit less well integrated: the Jean Bennett section reads like a detour, and the Hayles section is also unclear in its contribution to this specific paper. They need to either be unpacked more or maybe go altogether if the authors agree that situational analysis is a relevant background to be incorporated (which would require quite some space).

 My final remark is that I would not call this "method agnostic" as the authors do several times throughout the paper. True, the situational approach can be applied to different kinds of situations, but it has a very clear ANT inspiration that would certainly not be considered neutral by most people in the Humanities.

If any, are all the source data and materials underlying the results available?

No source data required

Is the work original in terms of material and argument?

Yes

Is the work clearly and cogently presented?

Yes

Is the argument persuasive and supported by evidence?

Partly

Does the research article contribute to the cultural, historical, social understanding of the field?

Yes

Does it sufficiently engage with relevant methodologies and secondary literature on the topic?

Partly

Reviewer Expertise:

I am a media researcher, with speciality in reception, audience studies, entertainment media.

I confirm that I have read this submission and believe that I have an appropriate level of expertise to confirm that it is of an acceptable scientific standard, however I have significant reservations, as outlined above.

# Response

**Published:** 2024-03-15

**Authors:** Marianne Gunderson

**Affiliations:** Linguistic, Literary and Aesthetic Studies, University of Bergen, Bergen, Hordaland, Norway

Dear Prof. Tosca,

 Thank you for this thorough reading and detailed review of our paper. We found your comments to be of great aid in our revisions and we believe that they have significantly contributed to the improvement of this text. We have made the following changes based on your feedback:

 In response to your comment on the cumulative aspects of the method, we have restructured some sections so that the quantitative results are highlighted in the conclusion and added a paragraph about the main findings from the database. We further clarified the relationship between the qualitative and quantitative contributions of this method by emphasising how the method also serves as an entry point to qualitative analysis of distributed agency.

 Regarding your query about Clarke's situational analysis, we agree that this was a glaring omission. We have added a paragraph discussing how the machine vision situation method relates to situational analysis, as well as how it could potentially supplement it. We have also included a paragraph emphasising the connection between our work and Haraway's concept of situated knowledges, which has been part of the theoretical backdrop for our work. Also, per your suggestion, we have shortened and integrated the Bennett section and moved the Hayles paragraph into the Mosaic Virus section, where its relevance should be more apparent.

 We agree that much more can be said about player or user agency in games and art, their absence in our analyses were an unforeseen result of our choice of examples. We have added a paragraph to both the art and games sections emphasising this and indicating how the user or player could come into play, as well as when and why we chose to include or exclude the player or user as an actor in our analyses. Here, we also point towards how the boundaries for which actors to include must be redrawn according to the priorities and limitations of the specific research project and indicate what this may bring out in the resulting analysis.

 The term 'method agnostic' was used to indicate that the method can be used both as a quantitative and a qualitative tool, but we have removed this term to avoid ambiguity.

 Finally, in response to your observations, we have sharpened the introduction by removing superfluous repetitions.

 We believe that we have addressed all your major objections and hope that this version will meet your expectations.
